# Evolutionary Signatures Governing the Codon Usage Bias in Coronaviruses and Their Implications for Viruses Infecting Various Bat Species

**DOI:** 10.3390/v13091847

**Published:** 2021-09-16

**Authors:** Naveen Kumar, Rahul Kaushik, Chandana Tennakoon, Vladimir N. Uversky, Anamika Mishra, Richa Sood, Pratiksha Srivastava, Meghna Tripathi, Kam Y. J. Zhang, Sandeep Bhatia

**Affiliations:** 1 Zoonotic Diseases Group, ICAR—National Institute of High Security Animal Diseases, Bhopal 462022, India; reach2anamika@yahoo.com (A.M.); richa.bhatia0609@gmail.com (R.S.); drpratikshasriv@gmail.com (P.S.); tripathimeghna0107@gmail.com (M.T.); sbhatia1967@gmail.com (S.B.); 2 Laboratory for Structural Bioinformatics, Center for Biosystems Dynamics Research, RIKEN, 1-7-22 Suehiro, Yokohama, Kanagawa 230-0045, Japan; rahul.kaushik@riken.jp (R.K.); kamzhang@riken.jp (K.Y.J.Z.); 3 Bioinformatics, Sequencing & Proteomics Group, The Pirbright Institute, Woking GU24 0NF, UK; chandana.tennakoon@pirbright.ac.uk; 4 Department of Molecular Medicine, Morsani College of Medicine, University of South Florida, Tampa, FL 33612, USA; vuversky@usf.edu; 5 Institute for Biological Instrumentation of the Russian Academy of Sciences, Federal Research Center ‘Pushchino Scientific Center for Biological Research of the Russian Academy of Sciences’, Moscow Region, 142290 Pushchino, Russia

**Keywords:** bats, coronaviruses, codon usage, CpG dinucleotide, evolution, SARS-CoV-2

## Abstract

Many viruses that cause serious diseases in humans and animals, including the betacoronaviruses (beta-CoVs), such as SARS-CoV, MERS-CoV, and the recently identified SARS-CoV-2, have natural reservoirs in bats. Because these viruses rely entirely on the host cellular machinery for survival, their evolution is likely to be guided by the link between the codon usage of the virus and that of its host. As a result, specific cellular microenvironments of the diverse hosts and/or host tissues imprint peculiar molecular signatures in virus genomes. Our study is aimed at deciphering some of these signatures. Using a variety of genetic methods we demonstrated that trends in codon usage across chiroptera-hosted CoVs are collaboratively driven by geographically different host-species and temporal-spatial distribution. We not only found that chiroptera-hosted CoVs are the ancestors of SARS-CoV-2, but we also revealed that SARS-CoV-2 has the codon usage characteristics similar to those seen in CoVs infecting the *Rhinolophus* sp. Surprisingly, the envelope gene of beta-CoVs infecting *Rhinolophus* sp., including SARS-CoV-2, had extremely high CpG levels, which appears to be an evolutionarily conserved trait. The dissection of the furin cleavage site of various CoVs infecting hosts revealed host-specific preferences for arginine codons; however, arginine is encoded by a wider variety of synonymous codons in the murine CoV (MHV-A59) furin cleavage site. Our findings also highlight the latent diversity of CoVs in mammals that has yet to be fully explored.

## 1. Introduction

Bats belong to the *Chiroptera* order, which is the second largest mammalian order after rodents and is the natural reservoir host for many key emerging and re-emerging viruses that cause serious human diseases [1,2,3]. Despite the fact that several of these viruses are lethal to humans, they are not known to produce clinical illnesses in bats. Megabats, which are mostly frugivorous (fruit eaters), and microbats, who are mostly insectivorous (insects-eater), are the two suborders of bats. Bats have lately been divided into two suborders based on molecular studies: *Yinpterochiroptera* (which includes megabats and several microbat species) and *Yangochiroptera* (which includes mostly microbat species) [4]. In addition to henipaviruses (Hendra and Nipah viruses) causing severe and often fatal diseases [5], coronaviruses (CoVs) are among the several chiroptera-borne viruses that have recently attracted the attention of the general and scientific communities.

CoVs are members of the *Orthocoronavirinae* subfamily of the *Coronaviridae* family and are divided into four genera: *Alpha*-, *Beta*-, *Gamma*-, and *Deltacoronaviruses* [6]. Increasing evidence suggests that bats are the reservoir host for alpha- and beta-CoVs that can infect a wide range of mammals, including humans, but gamma- and delta-CoVs are mostly found in birds [7]. The chiroptera-borne alpha- and beta-CoVs have been subclassified into eight subgenera (*Myotacovirus, Rhinacovirus, Setracovirus, Nyctacovirus, Minunacovirus, Decacovirus, Colacovirus*, and *Pedacovirus*), and four subgenera (*Nobecovirus, Merbecovirus, Hibecovirus,* and *Sarbecovirus*), respectively [8]. Fifth subgenera in beta-CoVs, the *Merbecovirus*, harbors murine CoVs, Myodes coronavirus 2JL14, Human coronavirus HKU1, and China Rattus coronavirus HKU24 [9]. Because RNA viruses have unique mechanism of replication and the intrinsically high potential for recombination and mutation, CoVs are recognized for rapidly adapting to new hosts and ecological niches [10,11,12,13]. The quest for CoVs in bats and other mammals has become a topic of global scientific and public interest with the appearance of SARS in 2002, MERS in 2012, and most recently COVID-19 in 2019.

Viruses are subjected to host-driven evolutionary pressures due to their entire reliance on host cellular machinery for their survival, and hence carry evolutionary signatures of their host micro-cellular milieu [14,15]. These evolutionary signatures make the viruses genetically and biochemically adapted to circulate in their hosts, in particular, reservoir hosts [16]. Furthermore, these viral evolutionary signatures have been revealed to influence a variety of biological processes in their hosts [17,18]. Deciphering these signatures is of particular interest for gaining insights into the evolutionary processes of synonymous codon usage and host-adapted evolution.

Although there are a plethora of genetic tools to study the evolutionary processes of synonymous codon usage and host-adapted evolution, we chose genetic tools that truly reflect evolution of signatures in virus genomes as a function of host tissues or cellular microenvironment, such as CpG dinucleotide contents and relative synonymous codon usage (RSCU). To better understand these evolutionary signatures, we combined and compared genome-level codon biases of chiroptera-hosted CoVs, seven human coronaviruses (HCoV-NL63, HCoV-229E, HCoV-HKU1, HCoV-OC43, MERS-CoV, SARS-CoV, and SARS-CoV-2), and representative *Gammacoronavirus* (Avian infectious bronchitis virus) and *Deltacoronavirus* (Porcine coronavirus). In order to understand the origin and evolution of SARS-CoV-2, we also looked for these signatures in the structural proteins encoded by CoVs infecting different hosts.

## 2. Materials and Methods

### 2.1. Coronavirus Genome Data Set

The full genomes of all chiroptera-hosted CoVs, alpha-CoVs (*n* = 93), beta-CoVs (*n* = 78) and unclassified-CoVs (*n* = 25) were retrieved from the NIAID Virus Pathogen Database and Analysis Resource (ViPR) through the web site at http://www.viprbrc.org/ (accessed on 11 July 2021) [19]. We also included seven human coronaviruses (HCoVs): two alpha-CoVs (HCoV-NL63 and HCoV-229E), and five beta-CoVs (HCoV-HKU1, HCoV-OC43, MERS-CoV, SARS-CoV, and SARS-CoV-2) to understand their evolutionary relationship with chiroptera-hosted CoVs. In addition, the final data set included representative gamma-CoV (Avian infectious bronchitis virus) and delta-CoV (Porcine coronavirus). Table S1 lists the demographics of all the CoVs used in this study. Because the genomic compositions of all four genera differ, open reading frames (ORFs) were concatenated from 5’ to 3’ to obtain full coding sequences for each strain. However, not all ORFs have been annotated; we chose four ORFs (S, E, M, and N) that are shared by all CoVs in order to learn more about their codon usage patterns and host-adapted molecular fingerprints.

### 2.2. Maximum-Likelihood Analysis

To assign genera/subgenera to the unclassified chiroptera-hosted CoVs and to understand the relationship of chiroptera-hosted CoVs with that of human and animal COVs, the complete coding sequences of CoVs from diverse host species were aligned using MAFFT v.7.475 [20] and a phylogenetic tree was constructed using the maximum likelihood statistical method with GTR-Gamma model in RAxML v. 8.2.12 [21]. In addition, evolutionary diversity and divergence between and within the chiroptera-host CoVs subgenera using the maximum composite likelihood model were also estimated [22]. The number of base substitutions per site between sequences was used to assess the evolutionary distance between them, or in other words, the sequence similarity. Besides, the number of base substitutions per site from averaging over all sequence pairs within each group and between the groups, respectively, is represented as the evolutionary divergence within and between the groups.

The mean evolutionary diversity for the entire population (${\hat{\pi }}_{T}$) was estimated as follows:

(1) $$\overset{}{{\hat{\pi }}_{T}=}\frac{q}{q-1}\sum_{}^{q}{\bar{X}}_{i}{\bar{X}}_{j}{\hat{d}}_{ij}$$

where *q* is the total number of alleles examined, ${\bar{X}}_{i}$ and ${\bar{X}}_{j}$ are the estimate of average frequency of the *i*-th and *j*-th alleles, respectively in the entire population, and ${\hat{d}}_{ij}$ is the frequency of nucleotide substitutions per site between the *i*-th and *j*-th alleles. The mean evolutionary diversity within subpopulations (${\hat{\pi }}_{S}$) was calculated as follows:

(2) $$\overset{}{{\hat{\pi }}_{S}=}\sum_{k=1}^{s}w_{k}{}_{}{\hat{\pi }}_{k}$$

where *s* represents the subpopulations, the relative size of the *k*-th subpopulation is $w_{k}$, and ${\hat{\pi }}_{k}$ is the estimate of average nucleotide diversity in the *k*-th subpopulation. The interpopulational evolutionary diversity (${\hat{\delta }}_{ST}$) was estimated using the following equation:

(3) $${\hat{\delta }}_{ST}={\hat{\pi }}_{T}-{\hat{\pi }}_{S}$$

### 2.3. Dinucleotide Composition Analysis

The dinucleotide frequencies for all the complete coding sequences of CoVs including the individual ORFs were calculated using Sequence Editor, Database, and Analysis Platform (SSE version 1.4) [23]. In addition, relative abundance (RA) of a dinucleotide was estimated as a ratio of observed to expected dinucleotide frequencies (assuming random association of mononucleotides from the observed frequencies of each mononucleotide for every sequence) implemented in DAMBE [24]. RA values of greater than 1.25 and less than 0.78 were defined as over- and under-representation, respectively [25].

(4) $$RA_{\left(XY\right)}=\frac{f_{XY}}{f_{X}*f_{Y}}$$

where ƒ*_X_* and ƒ*_Y_* are the frequencies of *X* and *Y* mononucleotide, respectively, and ƒ*_XY_* is the frequency of dinucleotide *XY*.

### 2.4. Relative Synonymous Codon Usage Analysis

The ratio of a codon’s observed frequency to its expected frequency, assuming that all codons encoding a particular amino acid are used equally, is known as RSCU [26]. To establish the patterns of synonymous codon usage without the confounding influence of amino acid compositions or sequence length, the RSCU values for complete coding sequences of all the CoVs used in this study including individual ORFs encoding sequences were determined. The RSCU values were calculated as follows:

(5) $$RSCU=\frac{g_{ij}}{\sum_{j}^{n_{i}}g_{ij}}n_{i}$$

where *g_ij_* is the observed number of the *i*th codon for the *j*th amino acid, which has *n_i_* kinds of the synonymous codons. Negative codon usage bias (less abundant codons) is represented by synonymous codons with RSCU values less than 1.0, while positive codon usage bias is represented by synonymous codons with RSCU values more than 1.0. (abundant codons). When RSCU = 1, it means that all synonymous codons for a given amino acid are used equally (no codon bias). Additionally, synonymous codons were divided into two groups based on their RSCU values: over-represented (RSCU > 1.6) and under-represented (RSCU < 0.6) codons [27].

### 2.5. Statistical Analysis

To analyze trends in codon use changes, the multivariate statistical method of correspondence analysis (COA) is often utilized. By eliminating discrepancies induced by uneven amino acid usage, the degrees of freedom were reduced to 40 (from 59 synonymous codons) when generating a correspondence analysis of RSCU [28]. Using relative inertia measurements and viral strains sorted along the key inertia axes, the main trends in the dataset were computed. COA was performed using the RSCU values of the full coding sequences CoVs, including the four ORFs (S, E, M, and N). The closest neighbors of a specific point were assessed using Euclidean distance, which measures the distance of two real-valued vectors or points across the COA axes. GraphPad Prism 7.01 was used for statistical analysis (GraphPad Software, San Diego, CA, USA). To assess the variations in evolutionary distances across the subgenera of chiroptera-hosted CoVs, we performed multiple *t*-tests with adjustments of the Holm–Sidak method. Dunn’s multiple comparison tests, a non-parametric test, were also used to assess the frequencies of synonymous codons encoding for the arginine amino acid. In these statistical studies, a *p*-value of less than 0.01 was considered statistically significant. All of the graphs were made with GraphPad Prism 7.01 software.

## 3. Results

### 3.1. The Chiroptera-Hosted Coronaviruses (CoVs) Have a Huge Evolutionary Diversity

**Figure 1:**
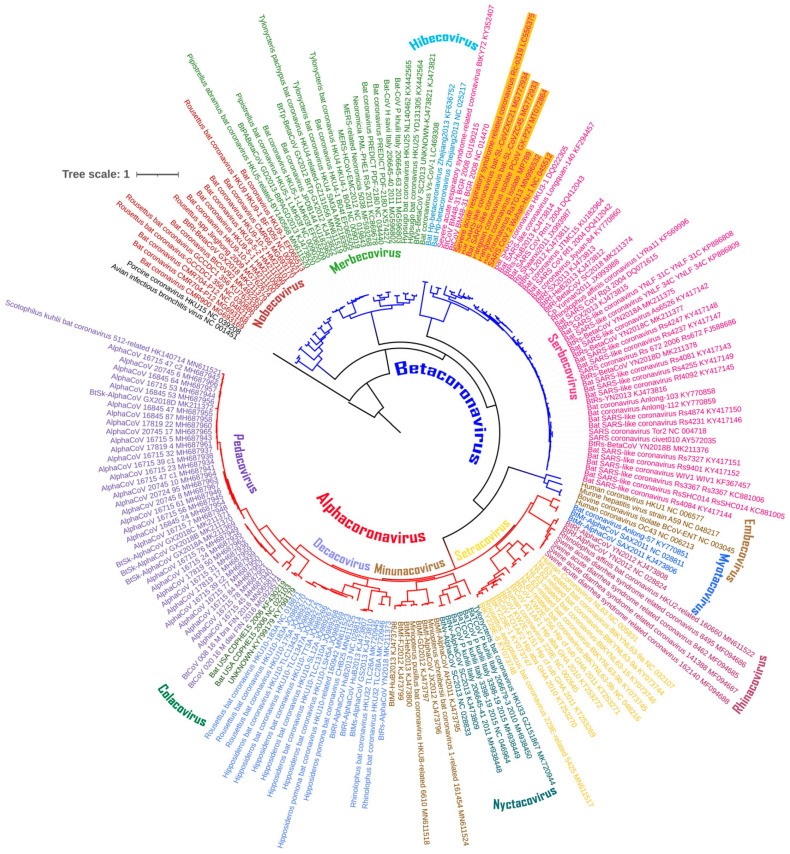
Maximum-likelihood (ML) tree showing the phylogenetic relationships based on the complete coding genomic sequences of coronaviruses (CoVs) infecting chiroptera, humans and different animals. The viruses are classified into genera and subgenera, and are colored differently. The tree was rooted to Avian Infectious Bronchitis virus, a gamma-CoV.

The chiroptera-hosted CoVs were first divided into subgenera, and then their evolutionary diversity was estimated (Figure 1). The mean evolutionary diversity (d ± SE) of all the chiroptera-hosted CoVs was 0.61 ± 0.15 indicating a huge diversity among these CoVs. In addition, we found that the mean evolutionary diversity between and within the subgenera of the chiroptera-hosted CoVs was 0.44 ± 0.11 and 0.16 ± 0.03, respectively.

**Figure 2:**
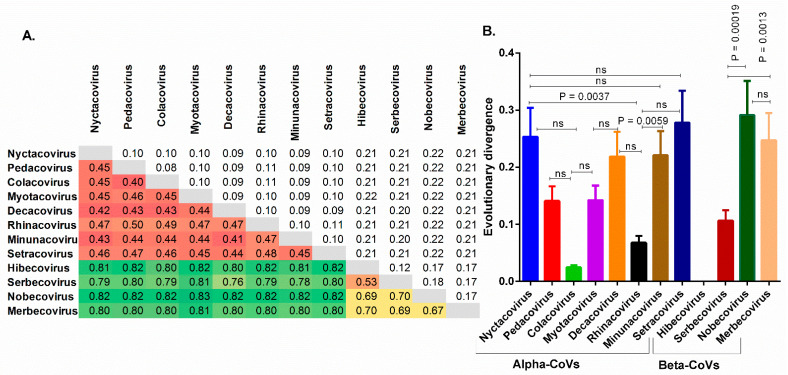
(**A**) shows a matrix showing the evolutionary divergence among the different subgenera of chiroptera-hosted CoVs. (**B**) shows a comparison of mean evolutionary divergence between the different subgenera of chiroptera-hosted CoVs. A *p*-value less than 0.01 is considered statistically significant, and ns represents non-significant difference between two values.

The evolutionary divergence of CoVs belonging to different subgenera of alpha- and beta-CoVs was also estimated. We found that the evolutionary divergence (d ± SE) among the eight subgenera of alpha-CoVs ranged from 0.39 ± 0.08 to 0.49 ± 0.11, while for beta-CoVs, it ranged from 0.52 ± 0.12 to 0.67 ± 0.16 among the four subgenera (Figure 2A). Furthermore, among alpha-CoVs, CoVs from *Nyctacovirus* and *Minunacovirus* have a statistically higher mean evolutionary divergence than CoVs from *Rhinacovirus* (*p* < 0.01), whereas in beta-CoVs, CoVs from *Serbecovirus* have the least mean evolutionary divergence compared to CoVs from *Nobecovirus* and *Merbecovirus* (*p* < 0.01 to 0.001) (Figure 2B). Notably, RaTG13, a *Serbecovirus* with a complete coding sequence identity of 96.8%, is the first closest match of SARS-CoV-2 among all the chiroptera-hosted, human and other animal-hosted CoVs followed by PCoV-MP789 with a 90.4% identity. These findings show that the CoVs have a great deal of genetic variety that has yet to be fully investigated.

### 3.2. Geographically Distinct Host Species and Temporo-Spatial Distribution Cooperatively Drive Trends in Codon Usage across Chiroptera-Hosted CoVs

**Figure 3:**
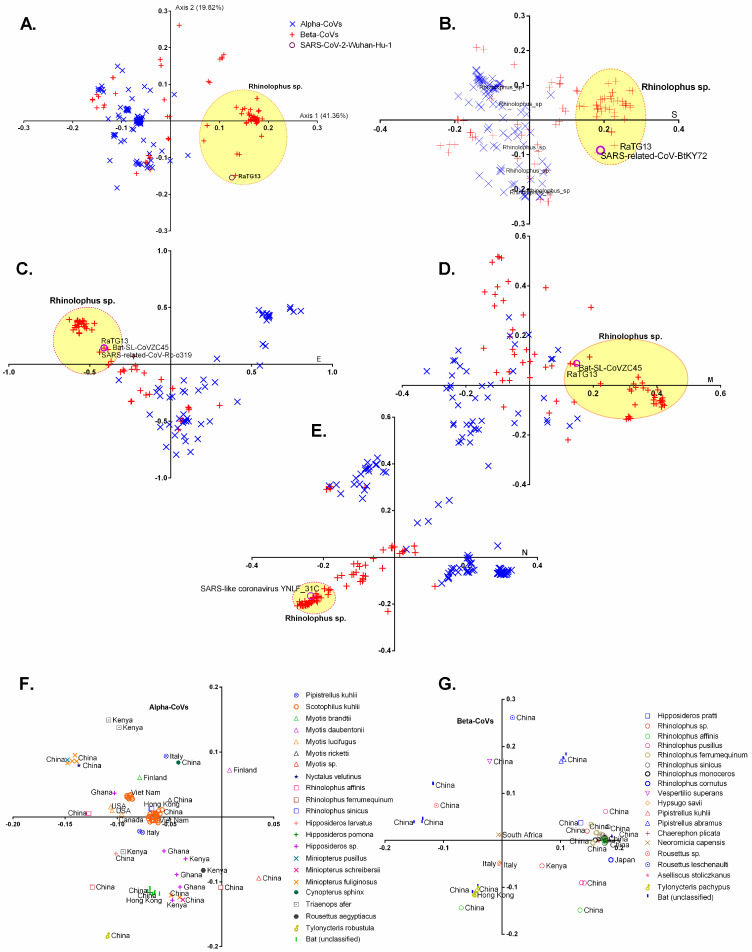
Correspondence analyses based on relative synonymous codon usage in the entire coding genomic sequences (**A**), spike (**B**), envelope (**C**), membrane (**D**), and nucleo-capsid (**E**) encoding sequences of chiroptera-hosted alpha- and beta-CoVs. (**F**,**G**) show trends in relative synonymous codon usage among CoVs infecting various bat species across different geographical locations. The viruses infecting *Rhinolophus* sp. are encircled and yellow-colored.

The RSCU values were utilised to analyse changes in synonymous codon usage in the full genome coding sequences of chiroptera-hosted CoVs as well as in the four ORFs (S, E, M, and N) that are shared by all CoVs using a multivariate statistical tool called correspondence analysis (COA). The very first ((ƒ’1, 41.36 percent) and second (ƒ’2, 19.82 percent) primary axis accounted for the majority of data inertia. The alpha-CoVs were separated from the beta-CoVs by the second (ƒ’2) principal axis; however, numerous beta-CoVs overlapped with alpha-CoVs (Figure 3A).

This is primarily due to the host species specific-adapted codon usage, for example, a majority of CoVs harbouring the *Rhinolophus* sp. clustered together, indicating the utilization of similar codon usage patterns (Figure 3A–E). Nevertheless, in addition to diverse host species, variations in the codon usage were also noted over the period of time and geographical distribution (Figure 3F,G).

**Figure 4:**
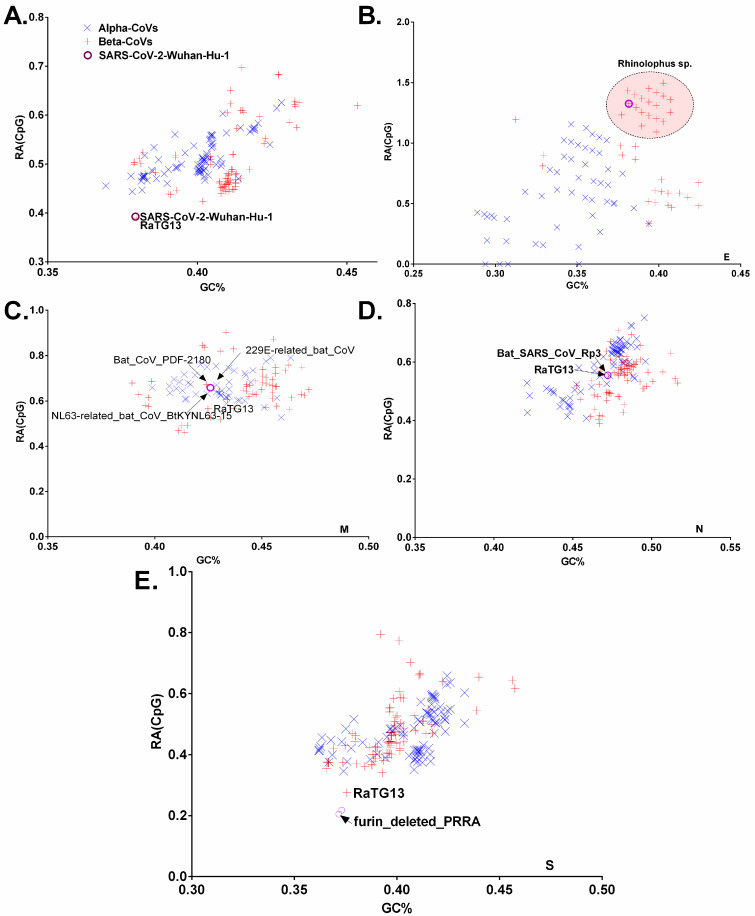
A plot of virus genomic RA_CpG_ against GC% in the entire coding genomic sequences (**A**), envelope (**B**), membrane (**C**), nucleo-capsid (**D**), and spike (**E**) encoding sequences of chiroptera-hosted alpha- and beta-CoVs. The closest matches with SARS-CoV-2 based on the shortest Euclidean distance are shown. The viruses infecting *Rhinolophus* sp. are red-encircled in Figure 4B, and SARS-CoV-2 is represented by a small purple circle.

The complete genome codon usage pattern of SARS-CoV-2 is the closest to RaTG13 (Euclidean distance or d = 0.0097) (Figure 3A). However, the codon usage patterns of E, M, N, and S protein of SARS-CoV-2 are closely clustered with RaTG13 (d = 0.0021), bat-SL-CoVZC45 (d = 0.0070), SARS-like coronavirus YNLF_31C (d = 0.0098), and SARS-related-CoV-BtKY72 (d = 0.0094), respectively, indicating that RaTG13 cannot be a recent ancestor of SARS-CoV-2, but SARS-CoV-2 carried codon usage signatures typically shown by CoVs infecting *Rhinolophus* sp. (Figure 3B–E). This finding is in line with a recent study that suggested an intermediate host in the zoonotic transmission of SARS-CoV-2 from bats to humans [16]. In addition, we analysed variations in synonymous codon usage in the complete genome coding sequences of different animal-hosted CoVs including humans as well as in the four ORFs (S, E, M, and N). We found that the complete genome codon usage patterns of SARS-CoV-2 remain the closest to RaTG13 and closely clustered with the ORFs of RaTG13, except spike (Figure 4A–E). These findings further support the idea that SARS-CoV-2 derived peculiar codon usage patterns from CoVs infecting bats.

### 3.3. Chiroptera-Hosted CoVs Were Identified as the Ancestor of SARS-CoV-2 in a Plot of Viral Genomic Relative Abundance of CpG (RA_CpG_) against GC%

**Figure 5:**
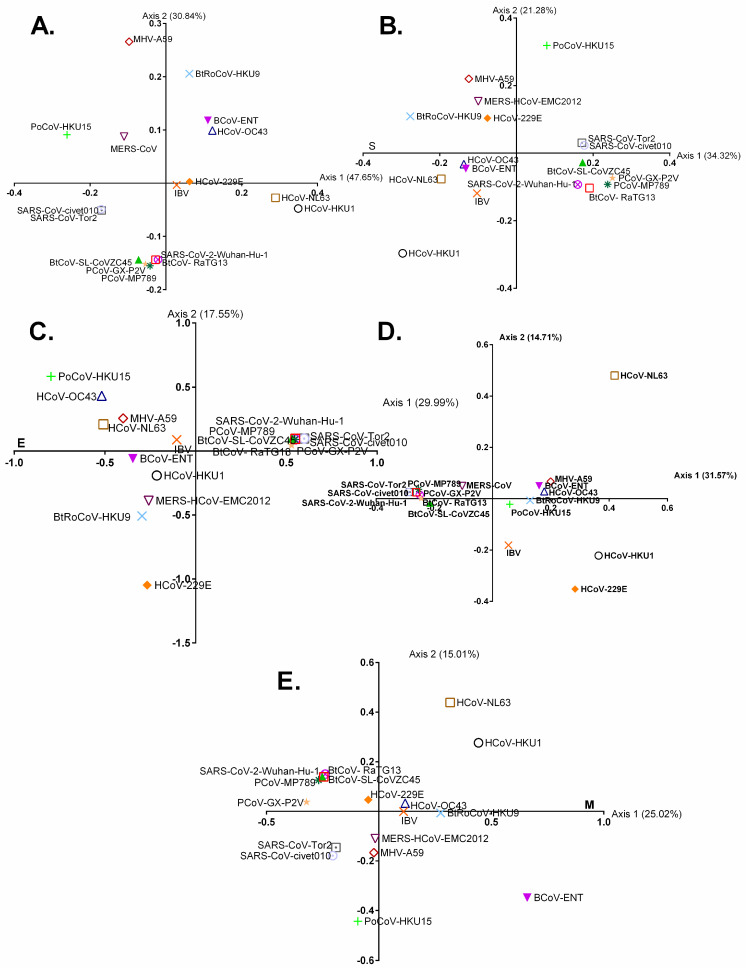
Correspondence analyses based on relative synonymous codon usage in the entire coding genomic sequences (**A**), spike (**B**), envelope (**C**), nucleocapsid (**D**), and membrane (**E**) encoding sequences of CoVs infecting diverse hosts.

The mean ± standard error of the mean (SEM) values for relative abundance of CpG (RA_CpG_) in the full coding genomic sequences of chiroptera-hosted alpha- and beta-CoVs are 0.513 ± 0.004, and 0.511 ± 0.008 respectively, which are significantly lower than the remaining dinucleotides (*p* < 0.01 to 0.0001). This CpG deficiency, however, is not significantly different between the alpha- and beta-CoVs (*p* > 0.01) (Figure S1). Since RA_CpG_ and genome GC% can differ between different viruses infecting the same host or the same virus infecting different hosts, we plotted virus genomic RA_CpG_ vs. GC% for the entire coding genomic sequences of alpha- and beta-CoVs including SARS-CoV-2. Importantly, SARS-CoV-2 and its closest phylogenetic relative RaTG13 grouped together (d = 0.0029) and exhibited the lowest RA_CpG_ (0.3925, and 0.3896, respectively) of all the chiroptera-hosted CoVs (Figure 5A). While looking at the ORFs, it is interesting to note that *Rhinolophus*-hosted beta-CoVs have unusually high RA_CpG_ in envelope genes, which appears to be evolutionarily conserved in this host, and the closest relative of SARS-CoV-2 membrane gene is SARS-related CoV- Rc-o319 (d = 0.00) (Figure 5B). The closest relatives of membrane protein are Bat_CoV_PDF_*Merbecovirus* (d = 0.0147) and 229E-related_bat_CoV_*Setracovirus* (d = 0.0123), while it shows Euclidean distance of 0.0934 with RaTG13 (Figure 5C). The closest relative of the nucleocapsid protein of SARS-CoV-2 is Bat_SARS_CoV_Rp3_2004_*Serbecovirus* (d = 0.0029) and, it shows Euclidean distance of 0.0309 with RaTG13 (Figure 5D). Even when compared to its closest phylogenetic relative RaTG13 (d = 0.0575), the spike gene of SARS-CoV-2 carried extremely low RA_CpG_ (0.2180) of all the chiroptera-hosted CoVs. The RA_CpG_ (0.2052) was further reduced by removing the furin cleavage site from the SARS-CoV-2 spike gene (Figure 5E).

**Figure 6:**
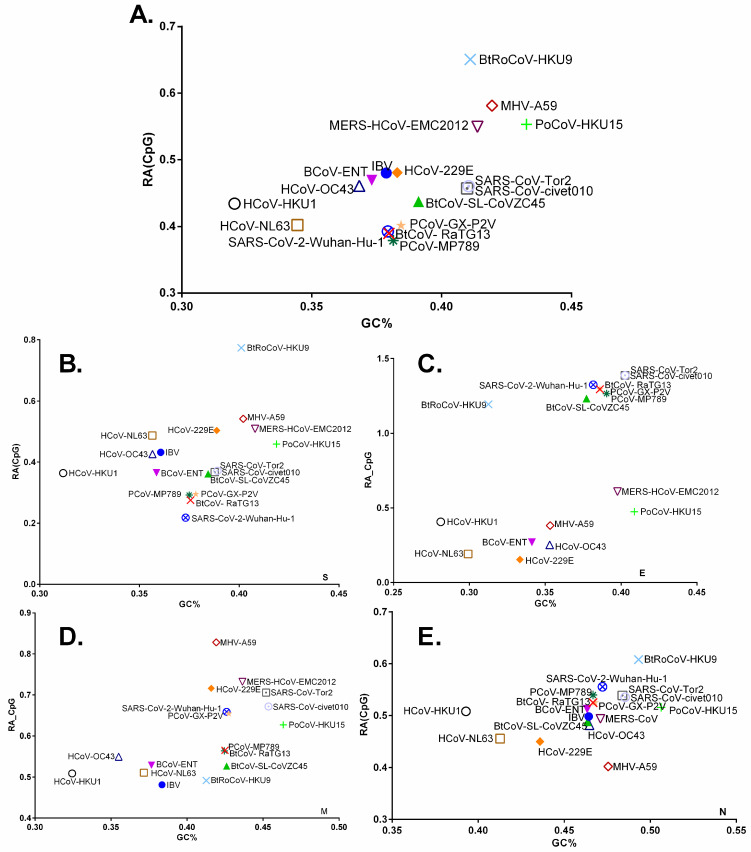
A plot of virus genomic RA_CpG_ against GC% in the entire coding genomic sequences (**A**), spike (**B**), envelope (**C**), membrane (**D**), and nucleocapsid (**E**) encoding sequences of CoVs infecting diverse hosts.

We also investigated the relationship between RA_CpG_ and genome GC% in human, avian, bovine, pangolins, civets, mouse, and porcine CoVs. Based on the entire genome, RaTG13 was found to be the closest relative of SARS-CoV-2 (d = 0.0029) (Figure 6A). The spike gene of SARS-CoV-2 demonstrated extreme RA_CpG_ deficiency among different animals that hosted CoVs, with RaTG13 (d = 0.0575) being the most closely related to SARS-CoV-2 among other animal CoVs (Figure 6B). Based on the envelope gene, RaTG13 and PCoV-GX-P2V (d = 0.0302) were at equal distance from SARS-CoV-2 (Figure 6C). The membrane and nucleocapsid of SARS-CoV-2, on the other hand, grouped closely with PCoV-GX-P2V (d = 0.0048) and PCoV-MP789 (d = 0.0167), respectively (Figure 6D,E).

### 3.4. Relative Synonymous Codon Usage (RSCU) Analysis

Without taking amino acid compositions into consideration, the RSCU analysis establishes synonymous codon usage patterns. As a result, we examined the RSCU values of chiroptera-hosted CoVs, human-hosted CoVs, and animal-hosted CoVs. We noted that, among the eight codons having CpG contents (GCG, CCG, CGA, CGC, CGG, CGU, UCG, ACG), six codons were under-represented, GCG(A), CCG(P), CGA(R), CGG (R), UCG (S), and ACG (T) in all animals and human-hosted CoVs, a peculiar feature of mammalian-adapted viruses.

**Figure 7:**
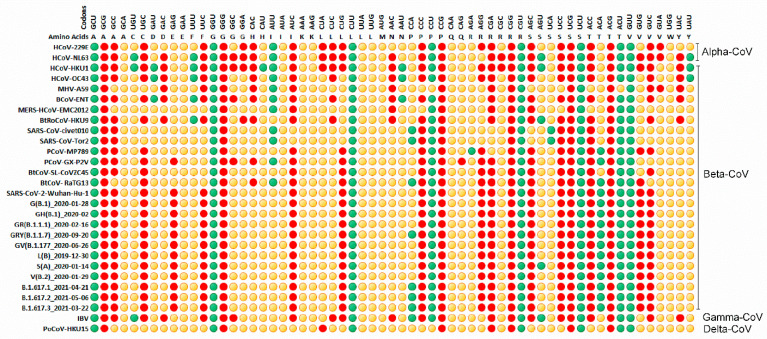
The relative synonymous codon usage in the full genomic coding sequences across the four genera of CoVs infecting diverse hosts. The synonymous codons were divided into three groups based on their relative synonymous codon usage (RSCU) values: RSCU > 1.6, green colored; RSCU < 1.6 to >0.6, yellow colored; and RSCU < 0.6, red colored.

Over-represented codons were those ending with U- GCU (A), GGU (G), CUU (L), CCU (P), CGU (R), UCU (S), ACU (T), GUU (V). The subgenera-specific usage of codons was observed, for example, under-representation of AAC(N) in *Rhinacovirus* and *Nobecovirus* of bat-CoVs, as well as in bovine, murine, human, and avian species hosted CoVs (Figure 7; Table S2). Furthermore, most *Serbecoviruses*, with the exception of pangolin-MP789, BtKY72, Rc-o319, and SARS-CoV-2 carry over-presentation of AUU(I) (Table S2).

Under-representation of CAC(H) and over-representation of AUU(I) in human, bovine, murine, and RaTG13 CoVs, but not in SARS-CoV-2, suggest that SARS-CoV-2 possesses peculiar synonymous codons usage (Figure 7). In addition, it is interesting to note that CAC(H) is under-represented in a majority of subgenera of chiropteran-hosted CoVs except *Serbecovirus*. Among the members of *Serbecovirus* including the SARS-CoV-2, only RaTG13 and BtKY72 carried under-represented CAC(H) (Table S2). The fact that CAC(H) is underrepresented and AUU(I) is overrepresented in human, bovine, murine, and RaTG13 CoVs but not in SARS-CoV-2 suggests that SARS-CoV-2 uses synonymous codons differently (Figure 7). Furthermore, with the exception of *Serbecovirus*, CAC(H) is under-represented in the majority of subgenera of chiroptera-hosted CoVs. Only RaTG13 and BtKY72 carried under-represented CAC(H) among *Serbecovirus* members, including SARS-CoV-2 (Table S2).

Importantly, under-representation of GAG(E) was found in SARS-CoV-2 and PCoV_GX-P2V only, and it persists in all SARS-CoV-2 lineages, implying that GAG under-representation is evolutionarily stable in SARS-CoV-2. Even the SARS-CoV-2 lineages have evolved to favour a particular codon, as evidenced by the over-representation of CCA(P) by B.1.617.1, B.1.617.2, B.1.617.3, and B.1.1.7, and AGU (S) by S(A) (Figure 6).

### 3.5. Dissection of the Furin Cleavage Site in CoVs Infecting Several Hosts

**Figure 8:**
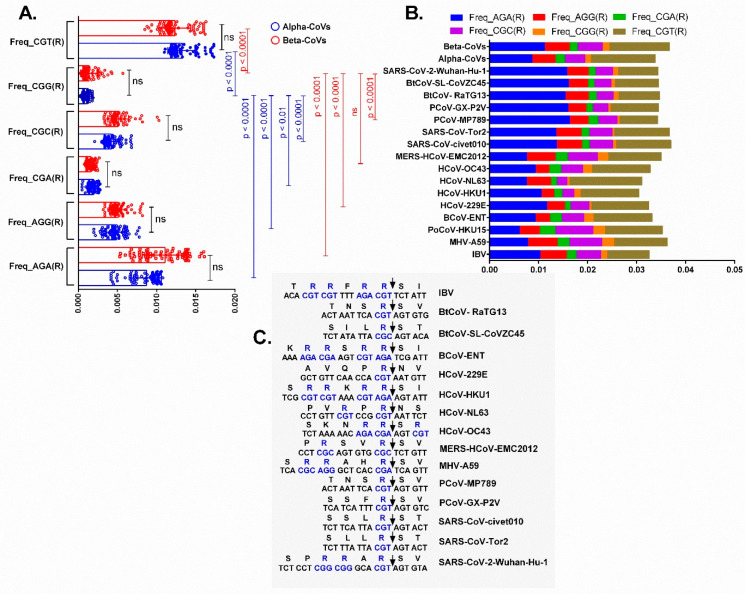
Frequency of arginine encoding synonymous codons in the complete genomic coding sequences of (**A**) chiroptera-hosted alpha- and beta-CoVs, (**B**) animals and humans-hosted CoVs. (**C**) shows the distribution patterns of arginine-encoding codons in the furin-like cleavage sites of CoVs infecting different hosts. A *p*-value less than 0.01 is considered statistically significant, and ns represents non-significant difference between two values.

We calculated the frequency of six arginine-coding codons (AGA, AGG, CGA, CGC, CGG, and CGT) across the entire coding sequences of chiroptera-hosted alpha- and beta-CoVs, including typical genera- and host-specific CoVs. CGG is the least favoured codon among all six codons (*p* < 0.01 to <0.0001), and that holds true for all chiroptera-borne CoVs. In chiroptera-hosted CoVs, however, CGT and AGA are the most often used codons encoding for arginine (*p* < 0.01 to <0.0001) (Figure 8A). In addition to bat-CoVs, we found host-specific preferences for arginine codons in complete coding CoV sequences. For example, most preferred codons encoding for arginine in avian-CoVs, IBV (AGA), murine, bovine and porcine CoVs (CGT), human-adapted CoVs (either CGT or AGA and CGT), MERS-CoV (CGT), SARS-CoV (AGA, and CGT), Pangolin-CoVs (either AGA or AGA and CGT), BtCoV- RaTG13 (AGA), BtCoV-SL-CoVZC45 (AGA, and CGT), and SARS-CoV-2-Wuhan-Hu-1 (AGA) were noted (*p* < 0.01 to <0.0001) (Figure 8B). Overall, AGA and CGT are the most frequently used codons among the CoVs, out of the six. Despite this host-specific variation in codon usage, CGG has consistently been found to be the least favoured codon (among the six arginine-coding codons) across CoVs infecting a variety of hosts, including bats (*p* < 0.01 to <0.0001).

Following that, we looked into each CoV’s furin-cleavage site in order to gain insight about the distribution patterns of arginine-encoding codons. CoVs found in avian, bovine, human (229E, HKU1, NL3, and OC43), pangolins, and bat often carry either CGT or CGT and AGA (Figure 8C). However, CGC (BtCoV-SL-CoVZC45, and MERS) as well as CGA (bovine) were also noted. Of note, in the murine CoV (MHV-A59) furin cleavage site, arginine is encoded by three different synonymous codons: CGC, AGG, and CGA. Finally, SARS-CoV-2 carried most frequently (CGT) and the least frequently used codons, two CGG placed together in its furin cleavage site.

## 4. Discussion

Virus genomes have unique evolutionary signatures as a result of adaptation to distinct hosts or micro-cellular environments. Understanding and elucidating these signatures in chiroptera-hosted CoVs are the prime interest of this study. Therefore, an investigation of these signatures at the complete genome- and ORFs-level of chiroptera-hosted CoVs was performed and compared with humans and animals-hosted CoVs to comprehend the role of these evolutionary signatures in the evolution of CoVs and host-adaptation. An unbiased search for these evolution signatures in SARS-CoV-2 and their comparison with other CoVs revealed that SARS-CoV-2 carried codon usage patterns and ancestral traits closely matching with chiroptera-hosted CoVs.

Increasing evidence suggests that bats are the reservoir host for alpha- and beta-CoVs [7,9,29,30,31]. The detection rates of both alpha- and beta-CoVs are substantially more in intestinal and faecal samples than throat or urine samples [12,32,33,34]. As a result, in spillover events or interspecies transmission of CoVs, bat excretion is the most common source of CoVs, for example, SARS-CoV and SARS-CoV-2 (*Serbecovirus*), MERS-CoV (*Merbecovirus*), and Swine Acute Diarrhea Syndrome coronavirus (SADS-CoV) (*Rhinacovirus*) [6,35,36,37]. Alpha-CoVs appear to be more prevalent than beta-CoVs, with a greater detection rate in bats [9]. However, mean evolutionary divergence among the members of beta-CoV subgenera is greater than that of alpha-CoV. It is worth noting that *Serbecovirus* and *Rhinacovirus* had the least mean evolutionary divergence among the beta-CoV and alpha-CoV subgenera, respectively. This is primarily due to detection of representatives of both subgenera in the same host, namely horseshoe bats, predominantly *Rhinolophus* sp., and therefore, they carried host-species specific adapted codon usage.

Next, on examining the trends in codon usage across the chiroptera-hosted CoVs, we found that codon usage patterns differed depending on host species and temporo-spatial distribution. Among all the chiroptera-hosted, human and other animals-hosted CoVs, the complete genome codon usage pattern of RaTG13 is the closest to SARS-CoV-2. Nevertheless, structural proteins of SARS-CoV-2 exhibited codon usage patterns similar to CoVs infecting *Rhinolophus* sp. Of note, the spike codon usage of SARS-CoV-2 is more comparable to SARS-related-CoV-BtKY72, detected in Kenya in 2007, implying that vast genetic diversity of CoVs in bats still remain largely unexplored. Furthermore, these findings show that SARS-CoV-2 had codon usage signatures comparable to those seen in CoVs that infect *Rhinolophus* sp., and that RaTG13 was unlikely to be its recent ancestor. We have yet to identify the CoVs that are more comparable to SARS-CoV-2 due to the latent diversity of CoVs. Notably, viruses (SARS-CoV, SARS-CoV-2, and Swine Acute Diarrhea Syndrome coronavirus, SADS-CoV) that have been responsible for major cross-species transmission events originated from *Rhinolophus* sp. Globally, *Rhinolophus* sp. is distributed primarily in Asia, Europe and Africa, with the most diversity of bat SARS-like CoVs detected in China’s Yunnan Province, where many recombination events occurred [38,39]. As a result of our findings, enhanced screening of bats and wildlife for identifying viruses with zoonotic spillover potential is required. Our study could help predict future cross-species transmission occurrences based on the geographic distribution of bat species.

Many RNA viruses including CoVs exhibit strong CpG deficiency, which is caused by two mammalian enzymes, zinc finger antiviral protein (ZAP) and apolipoprotein B mRNA editing enzyme (APOBEC3G) [16]. In mammals, ZAP is a critical component of the interferon-mediated immune response, and its RNA-binding domain binds preferentially to CpG dinucleotides in viral RNA genomes [40]. Another antiviral enzyme found in innate immune cells, APOBEC3G, deaminates C to U (CpG to UpG) in RNA viruses, making them less vulnerable to CpG-mediated ZAP attack [41,42,43]. Both antiviral enzymes contribute to CpG deficiency in RNA viruses, and because these enzymes are expressed differentially in different organs, viruses infecting different tissues should have different CpG signatures. Therefore, to unravel these CpG signatures in chiroptera-hosted, human and animal hosted-CoVs, we plotted RA_CpG_ against GC% for the entire coding genomic sequences as well as different structural protein encoding sequences. While examining the entire coding genomic sequences, SARS-CoV-2 and its closest phylogenetic relative RaTG13 clustered together and more crucially, the spike gene of SARS-CoV-2 revealed extreme CpG deficiency among all chiroptera-hosted, human and animal hosted-CoVs. This shows that SARS-CoV-2 might have assimilated an extreme CpG deficiency in tissues or cells expressing high levels of ZAP and/or APOBEC3G.

Since different proteins encoded by a viral genome owing to different functions experience diverse selection pressure, we noted varied CpG contents in structural proteins of chiroptera, human and animal-hosted CoVs. Strikingly, the envelope gene of beta-CoVs infecting *Rhinolophus* sp., including SARS-CoV-2, had extremely high CpG contents, which appears to be an evolutionary conserved trait possessed by these CoVs. This finding is in line with the results of previous research, which revealed that high CpG conservation in the envelope gene has a role that overcomes the need to suppress CpG, and so could be advantageous in the development of an attenuated SARS-CoV-2 vaccine candidate [44]. Essentially, SARS-CoV-2 had CpG signatures that were similar to those found in chiroptera-borne CoVs.

The furin cleavage site carried by the spike protein of SARS-CoV-2 is the most contentious aspect of the genesis of this virus. The unique furin cleavage site (RRAR) in the spike protein of SARS-CoV-2 is responsible for its pathogenesis [45] and enhanced transmissibility [46]. We found host-specific preferences for arginine codons in the entire CoV coding sequences, with AGA and CGT being the most favoured codons, and CGG being the least favoured codons in the diverse hosts, based on an unbiased investigation into the furin-like cleavage site in CoVs infecting diverse hosts. These observations remain nearly the same in the furin cleavage site; i.e., the use of CGT or CGT and AGA in avian, bovine, human, pangolins, and bats (RaTG13, and RmYN02). RmYN02, which was recently detected in *Rhinolophus malayanus*, shares 93.3% of its genomic nucleotide identity with SARS-CoV-2, and contains a closely homologous sequence (PAAR) at the S1/S2 cleavage site; however, it lacks basic amino acids essential for cleavage by furin-like protease [47]. Nevertheless, together with the recent detection of a CoV in bats with a sequence that is comparable to that of the furin-like cleavage site, these observations underscore that the untapped diversity of CoVs in mammals remains to be investigated.

Importantly, arginine in the murine CoV (MHV-A59) furin cleavage site is encoded by a wider range of synonymous codons. The least preferred codons CGG (in duplicate) placed together in the furin cleavage site of SARS-CoV-2 have not been noticed in any of the CoVs investigated including the chiroptera-hosted CoVs. Given the extremely low CpG contents of SARS-CoV-2 spike protein compared to all other CoVs, including its closest phylogenetic relative RaTG13, we demonstrated that removing the furin cleavage site further reduced its total CpG contents, implying that the selection of codons carrying CpG component in the furin cleavage site overrides the need to reduce the CpG contents in the furin cleavage site of spike protein. In support of this, the recently identified SARS-CoV-2 delta variant has a crucial mutation (CCT to CGT) in its furin cleavage site, resulting in the insertion of an extra arginine, **P**RRARSVA (Wuhan) to **R**RRARSVA (delta variant). This introduction highlights host-adapted SARS-CoV-2 evolution by emphasising the preference of virus for the most frequently used codon (CGT).

The findings of this study suggest that the likelihood of direct zoonotic spillover of SARS-CoV-2 from bats to humans is unlikely, and that a critical intermediary host link is missing. These viruses are anticipated to emerge and re-emerge in the future, given the large diversity of CoVs infecting multiple bat species, as well as increased spillover events (directly or indirectly from bats to humans) primarily owing to ecological, behavioural, or socioeconomic changes. Therefore, coordinated surveillance efforts in identifying chiroptera-hosted viruses with zoonotic spillover potential and understanding ecological risk factors for transmission should be undertaken to aid in the deployment of preventive measures.

## Data Availability

Not applicable.

## Supplementary Materials

The following are available online at https://www.mdpi.com/article/10.3390/v13091847/s1, Figure S1: A comparison of relative abundance of CpG dinucleotides between the chiroptera-hosted alpha- and beta-CoVs, Table S1: Demographics of chiroptera-hosted coronaviruses used in this study, Table S2: Relative synonymous codon usage values for the complete coding genomic sequences of chiroptera-hosted CoVs.
