# Supplementary material for: Evolutionary Signatures Governing the Codon Usage Bias in Coronaviruses and Their Implications for Viruses Infecting Various Bat Species
Source: Viruses. 2021 Sep 16;13(9):1847. doi: 10.3390/v13091847 (PMC8473330; doi:10.3390/v13091847)
Supplement: Supplementary file 1 [file viruses-13-01847-s001.zip › Supplementary information captions.pdf]

Figure S1. A comparison of relative abundance of CpG dinucleotides between the chiroptera-hosted alpha- and beta-CoVs

Table S1. Demographics of chiroptera-hosted coronaviruses used in this study

Table S2 Relative synonymous codon usage values for the complete coding genomic sequences of chiroptera-hosted CoV
