# Supplementary material for: Evolutionary Signatures Governing the Codon Usage Bias in Coronaviruses and Their Implications for Viruses Infecting Various Bat Species
Source: Viruses. 2021 Sep 16;13(9):1847. doi: 10.3390/v13091847 (PMC8473330; doi:10.3390/v13091847)
Supplement: Supplementary file 1 [file viruses-13-01847-s001.zip › Table S1.pdf]

Table S1. Demographics of chiroptera-hosted coronaviruses used in this study

GenBank Accession	Strain Name	Sequence Length	Collection Date	GenBank Host	Country
MH938448	Bat-CoV/P.kuhlii/Italy/206645-41/2011	27862	2011	Pipistrellus kuhlii	Italy
MH938450	Bat-CoV/P.kuhlii/Italy/206679-3/2010	28146	2010	Pipistrellus kuhlii	Italy
MH938449	Bat-CoV/P.kuhlii/Italy/3398-19/2015	28128	2015	Pipistrellus kuhlii	Italy
NC_046964*	Bat-CoV/P.kuhlii/Italy/3398-19/2015	28128	2015	Pipistrellus kuhlii	Italy
MH687934	VZ_AlphaCoV_16715_23	28249	06/11/2014	Scotophilus kuhlii	Viet Nam
MH687935	VZ_AlphaCoV_16715_24	27757	06/17/2014	Scotophilus kuhlii	Viet Nam
MH687936	VZ_AlphaCoV_16715_31	28181	06/17/2014	Scotophilus kuhlii	Viet Nam
MH687937	VZ_AlphaCoV_16715_32	28236	06/17/2014	Scotophilus kuhlii	Viet Nam
MH687938	VZ_AlphaCoV_16715_39_c1	28349	06/17/2014	Scotophilus kuhlii	Viet Nam
MH687939	VZ_AlphaCoV_16715_39_c2	28307	06/17/2014	Scotophilus kuhlii	Viet Nam
MH687940	VZ_AlphaCoV_16715_45	28170	06/10/2014	Scotophilus kuhlii	Viet Nam
MH687941	VZ_AlphaCoV_16715_47_c1	28008	06/17/2014	Scotophilus kuhlii	Viet Nam
MH687942	VZ_AlphaCoV_16715_47_c2	28321	06/17/2014	Scotophilus kuhlii	Viet Nam
MH687943	VZ_AlphaCoV_16715_5	28240	06/10/2014	Scotophilus kuhlii	Viet Nam
MH687944	VZ_AlphaCoV_16715_53	28219	06/10/2014	Scotophilus kuhlii	Viet Nam
MH687945	VZ_AlphaCoV_16715_56	28235	09/16/2014	Scotophilus kuhlii	Viet Nam
MH687946	VZ_AlphaCoV_16715_61	28173	06/10/2014	Scotophilus kuhlii	Viet Nam
MH687947	VZ_AlphaCoV_16715_63	22363	06/17/2014	Scotophilus kuhlii	Viet Nam
MH687948	VZ_AlphaCoV_16715_7	28255	06/11/2014	Scotophilus kuhlii	Viet Nam
MH687949	VZ_AlphaCoV_16715_76	28185	06/10/2014	Scotophilus kuhlii	Viet Nam
MH687950	VZ_AlphaCoV_16715_77	28155	06/10/2014	Scotophilus kuhlii	Viet Nam
MH687951	VZ_AlphaCoV_16715_78	28313	06/11/2014	Scotophilus kuhlii	Viet Nam
MH687952	VZ_AlphaCoV_16715_84	27225	06/10/2014	Scotophilus kuhlii	Viet Nam

MH687953	VZ_AlphaCoV_16715_86	23258	06/11/2014	Scotophilus kuhlii	Viet Nam
MH687954	VZ_AlphaCoV_16845_24	28240	09/18/2014	Scotophilus kuhlii	Viet Nam
MH687955	VZ_AlphaCoV_16845_47	28219	09/17/2014	Scotophilus kuhlii	Viet Nam
MH687956	VZ_AlphaCoV_16845_53	28050	09/16/2014	Scotophilus kuhlii	Viet Nam
MH687957	VZ_AlphaCoV_16845_64	28173	09/18/2014	Scotophilus kuhlii	Viet Nam
MH687958	VZ_AlphaCoV_16845_87	28036	09/18/2014	Scotophilus kuhlii	Viet Nam
MH687959	VZ_AlphaCoV_17819_17	28169	11/12/2014	Scotophilus kuhlii	Viet Nam
MH687960	VZ_AlphaCoV_17819_22	27303	11/13/2014	Scotophilus kuhlii	Viet Nam
MH687961	VZ_AlphaCoV_17819_4	28168	11/13/2014	Scotophilus kuhlii	Viet Nam
MH687962	VZ_AlphaCoV_17819_50	27752	11/13/2014	Scotophilus kuhlii	Viet Nam
MH687963	VZ_AlphaCoV_20724_95	28169	02/06/2015	Scotophilus kuhlii	Viet Nam
MH687964	VZ_AlphaCoV_20745_10	28210	02/06/2015	Scotophilus kuhlii	Viet Nam
MH687965	VZ_AlphaCoV_20745_17	28115	02/13/2015	Scotophilus kuhlii	Viet Nam
MH687966	VZ_AlphaCoV_20745_6	28389	02/06/2015	Scotophilus kuhlii	Viet Nam
MH687967	VZ_AlphaCoV_20745_8	27726	02/06/2015	Scotophilus kuhlii	Viet Nam
MN065811	BtCoV/008_16/M.bra/FIN/2016	28119	08/15/2016	Myotis brandtii	Finland
MG923574	BtCoV/020_16/M.dau/FIN/2016	28045	08/28/2016	Myotis daubentonii	Finland
NC_022103*	bat/USA/CDPHE15/2006	28035	08/07/2006	Myotis lucifugus	USA
KY799179	UNKNOWN-KY799179	28173	11/2010	Myotis lucifugus	Canada
KJ473806	BtMr-SAX2011	27935	2011	Myotis ricketti	China
NC_028811*	BtMr-SAX2011	27935	2011	Myotis ricketti	China
KJ473809	BtNv-SC2013	27783	2013	Nyctalus velutinus	China
NC_028833*	BtNv-SC2013	27783	2013	Nyctalus velutinus	China
KJ473810	BtMs-GS2013	27576	2013	Myotis sp.	China
KJ473807	BtRf-HuB2013	27608	2013	Rhinolophus ferrumequinum	China
NC_028814*	BtRf-HuB2013	27608	2013	Rhinolophus ferrumequinum	China
KJ473808	BtRf-YN2012	26975	2012	Rhinolophus ferrumequinum	China

NC_028824*	BtRf-YN2012	26975	2012	Rhinolophus ferrumequinum	China
MK211373	BtRs-AlphaCoV/YN2018	29109	05/2017	Cynopterus sphinx	China
MK211369	BtSk-AlphaCoV/GX2018A	28303	05/2017	Scotophilus kuhlii	China
MK211370	BtSk-AlphaCoV/GX2018B	28175	05/2017	Scotophilus kuhlii	China
MK211371	BtSk-AlphaCoV/GX2018C	28146	05/2017	Scotophilus kuhlii	China
MK211372	BtSk-AlphaCoV/GX2018D	28238	05/2017	Scotophilus kuhlii	China
MN611525	CHB0025	28169	03/2018	Hipposideros larvatus	China
MN611523	160942	28514	03/2018	Hipposideros pomona	China
MN611518	6610	28751	03/2018	Miniopterus pusillus	China
MN611524	161454	28316	03/2018	Miniopterus schreibersii	China
KY073746	BtKYNL63-15	28442	08/06/2008	Triaenops afer	Kenya
KY073744	BtKYNL63-9a	28363	06/21/2010	Triaenops afer	Kenya
NC_032107*	BtKYNL63-9a	28363	06/21/2010	Triaenops afer	Kenya
KY073745	BtKYNL63-9b	28679	06/21/2010	Triaenops afer	Kenya
NC_048216*	BtKYNL63-9b	28679	06/21/2010	Triaenops afer	Kenya
MN611522	160660	26956	03/2018	Rhinolophus affinis	China
MK720945	TLC26A	29201	08/06/2015	Rhinolophus sinicus	Hong Kong
MK720946	TLC28A	29201	08/06/2015	Rhinolophus sinicus	Hong Kong
MN611517	5425	27619	03/2018	Rousettus aegyptiacus	Kenya
MN611521	HK140714	27933	03/2018	Scotophilus kuhlii	China
MK720944	GZ151867	27636	09/09/2015	Tylonycteris robustula	China
KJ473795	BtMf-AH2011	28310	2011	Miniopterus fuliginosus	China
KJ473796	BtMf-JX2012	28311	2012	Miniopterus fuliginosus	China
KF430219	bat/USA/CDPHE15/2006	28035	08/07/2006	Myotis lucifugus	USA
JQ989269	LSH5A	28492	12/2005	bat	Hong Kong
JQ989267	SL12A	28483	12/2006	bat	Hong Kong
JQ989268	TLC1310A	28483	10/2006	bat	Hong Kong

JQ989272	TLC1343A	28483	08/2010	bat	Hong Kong
JQ989273	TLC1347A	28483	08/2010	bat	Hong Kong
JQ989266	TT3A	28483	12/2006	bat	Hong Kong
JQ989271	175A	28489	10/2005	bat	China
JQ989270	183A	28494	10/2005	bat	China
NC_018871*	183A	28494	10/2005	bat	China
KT253272	BtCoV/AT1A-F1/Hip_aba/GHA/2010	28688	2010	Hipposideros abae	Ghana
KT253270	BtCoV/FO1A-F2/Hip_aba/GHA/2010	28020	2010	Hipposideros abae	Ghana
KT253269	BtCoV/KW2E-F151/Hip_cf._rub/GHA/2011	28026	2011	Hipposideros cf. ruber	Ghana
KT253271	BtCoV/KW2E-F56/Hip_cf._rub/GHA/2011	28754	2011	Hipposideros cf. ruber	Ghana
KY073747	BtKY229E-1	27837	06/20/2009	Hipposideros sp.	Kenya
KY073748	BtKY229E-8	27636	06/2010	Hipposideros vittatus	Kenya
KJ473799	BtMf-FJ2012	28765	2012	Miniopterus fuliginosus	China
KJ473797	BtMf-GD2012	28758	2012	Miniopterus fuliginosus	China
KJ473800	BtMf-HeN2013	28735	2013	Miniopterus fuliginosus	China
KJ473798	BtMf-HuB2013	28755	2013	Miniopterus fuliginosus	China
NC_025217*	Zhejiang2013	31491	04/29/2013	Hipposideros pratti	China
MK211374	BtRl-BetaCoV/SC2018	29648	08/2016	Rhinolophus sp.	China
MK211375	BtRs-BetaCoV/YN2018A	29698	09/2016	Rhinolophus affinis	China
MK211376	BtRs-BetaCoV/YN2018B	30256	09/2016	Rhinolophus affinis	China
MK211377	BtRs-BetaCoV/YN2018C	29689	09/2016	Rhinolophus affinis	China
MK211378	BtRs-BetaCoV/YN2018D	30213	09/2016	Rhinolophus affinis	China
MK211379	BtRt-BetaCoV/GX2018	29752	09/2016	Rhinolophus affinis	China
KJ473821	UNKNOWN-KJ473821	30423	-N/A-	Vespertilio superans	China
MG596802	Bat-CoV/H.savii/Italy/206645-40/2011	30048	2011	Hypsugo savii	Italy
MG596803	Bat-CoV/P.khulii/Italy/206645-63/2011	30039	2011	Pipistrellus kuhlii	Italy
MF593268	Neoromicia/5038	30009	04/20/2015	Neoromicia capensis	South Africa

MN611520	BY140568	30511	03/2018	Pipistrellus abramus	China
NC_030886*	GCCDC1 356	30161	05/28/2014	Rousettus leschenaulti	China
MT350598	GCCDC1	30162	10/2016	Eonycteris spelaea	Singapore
KT444582	WIV16	30290	07/21/2013	Rhinolophus sinicus	China
JX993988	Cp/Yunnan2011	29452	2011	Chaerephon plicata	China
JX993987	Rp/Shaanxi2011	29484	09/2011	Rhinolophus pusillus	China
KJ473812	BtRf-HeB2013	29443	2013	Rhinolophus ferrumequinum	China
KJ473811	BtRf-JL2012	29037	2012	Rhinolophus ferrumequinum	China
KJ473813	BtRf-SX2013	29461	2013	Rhinolophus ferrumequinum	China
KJ473815	BtRs-GX2013	29161	2013	Rhinolophus sinicus	China
KJ473814	BtRs-HuB2013	29658	2013	Rhinolophus sinicus	China
KJ473816	BtRs-YN2013	29142	2013	Rhinolophus sinicus	China
MN611519	GZ131656	30224	03/2018	Tylonycteris pachypus	China
KF636752	Zhejiang2013	31491	04/29/2013	Hipposideros pratti	China
KC869678	Neoromicia/PML-PHE1/RSA/2011	30111	2011	Neoromicia capensis	South Africa
EF065510	TT03f	30488	-N/A-	bat	China
EF065511	TT06f	30488	-N/A-	bat	China
EF065512	TT07f	30487	-N/A-	bat	China
EF065509	LMH03f	30482	-N/A-	bat	China
KJ473820	BtPa-GD2013	30480	2013	Pipistrellus abramus	China
NC_009020*	HKU5-1 LMH03f	30482	-N/A-	bat	China
EF065514	BF_017I	29107	-N/A-	bat	China
EF065515	BF_493I	29136	-N/A-	bat	China
EF065516	BF_141I	29155	-N/A-	bat	China
EF065513	BF_005I	29114	-N/A-	bat	China
HM211100	UNKNOWN-HM211100	29136	04/10/2006	bat BF_506I	China
HM211101	UNKNOWN-HM211101	29122	04/10/2006	bat BF_506I	China

HM211098	UNKNOWN-HM211098	29136	12/11/2005	bat BF_258I	China
HM211099	UNKNOWN-HM211099	29112	12/11/2005	bat BF_258I	China
NC_009021*	HKU9-1 BF_005I	29114	-N/A-	bat	China
MG762674	Rousettus spp/Jinghong/2009	29134	11/01/2009	Rousettus sp.	China
DQ022305	HKU3-1	29728	-N/A-	-N/A-	China
DQ412042	Rf1	29709	-N/A-	Rhinolophus ferrumequinum	-N/A-
DQ412043	Rm1	29749	-N/A-	Rhinolophus macrotis	-N/A-
DQ071615	Rp3	29736	-N/A-	bat	China
MN996532	RaTG13	29855	07/24/2013	Rhinolophus affinis	China
KY417142	As6526	29725	05/12/2014	Aselliscus stoliczkanus	China
MG772933	bat-SL-CoVZC45	29802	02/2017	Rhinolophus pusillus	China
MG772934	bat-SL-CoVZXC21	29732	07/2015	Rhinolophus pusillus	China
KF294457	Longquan-140	29676	2012	Rhinolophus monoceros	China
KY417145	Rf4092	29710	09/18/2012	Rhinilophus ferrumequinum	China
KY417143	Rs4081	29741	09/18/2012	Rhinolophus sinicus	China
KY417144	Rs4084	29770	09/18/2012	Rhinolophus sinicus	China
KY417146	Rs4231	29782	04/17/2013	Rhinolophus sinicus	China
KY417147	Rs4237	29741	04/17/2013	Rhinolophus sinicus	China
KY417148	Rs4247	29743	04/17/2013	Rhinolophus sinicus	China
KY417149	Rs4255	29743	04/17/2013	Rhinolophus sinicus	China
KY417150	Rs4874	30311	07/21/2013	Rhinolophus sinicus	China
KY417151	Rs7327	30307	10/24/2014	Rhinolophus sinicus	China
KY417152	Rs9401	29769	10/16/2015	Rhinolophus sinicus	China
KC881006	Rs3367	29792	03/19/2012	Rhinolophus sinicus	China
KC881005	RsSHC014	29787	04/17/2011	Rhinolophus sinicus	China
KF367457	WIV1	30309	09/2012	Rhinolophus sinicus	China
KP886808	YNLF_31C	29723	05/23/2013	Rhinolophus Ferrumequinum	China

KP886809	YNLF_34C	29723	05/23/2013	Rhinolophus Ferrumequinum	China
KF569996	LYRa11	29805	2011	Rhinolophus affinis	China
FJ588686	Rs672	29059	2006	Rhinolophus sinicus	China
KY352407	BtKY72	29274	08/2007	Rhinolophus sp. (bat)	Kenya
KU973692	F46	29722	2012	bat	China
LC556375	Rc-o319	29718	2013	Rhinolophus cornutus	Japan
EF065506	B05f	30286	-N/A-	bat	China
EF065507	B07f	30286	-N/A-	bat	China
EF065508	LMH1f	30316	-N/A-	bat	China
EF065505	B04f	30286	-N/A-	bat	China
KJ473822	BtTp-GX2012	30247	2012	Tylonycteris pachypus	China
NC_009019*	HKU4-1 B04f	30286	-N/A-	bat	China
MW218395	SM3A	30248	08/16/2010	Tylonycteris pachypus	Hong Kong
KX442565	NL140462	30497	05/08/2014	Hypsugo pulveratus	China
KX442564	YD131305	30498	09/14/2013	Hypsugo pulveratus	China
KU762338	GCCDC1 356	30161	05/28/2014	Rousettus leschenaulti	China
MF094687	141388	27174	-N/A-	Rhinolophus sp.	China
MF094688	162140	27177	-N/A-	Rhinolophus sp.	China
MF094685	8462	27200	-N/A-	Rhinolophus sp.	China
MF094686	8495	27198	-N/A-	Rhinolophus sp.	China
KY770858	Anlong-103	29688	2013	Rhinolophus sinicus	China
KY770859	Anlong-112	29667	2013	Rhinolophus sinicus	China
KY770850	Anlong-43	26883	2013	Myotis siligorensis	China
KY770851	Anlong-57	28096	2013	Myotis davidii	China
MK492263	BtCoV92	29585	2015	Cynopterus brachyotis	Singapore
MG693170	CMR66	29123	12/2013	Eidolon helvum	Cameroon
MG693168	CMR704-P12	28975	12/2013	Eidolon helvum	Cameroon

NC_048212*	CMR704-P12	28975	12/2013	Eidolon helvum	Cameroon
MG693172	CMR705-P13	28958	12/2013	Eidolon helvum	Cameroon
MG693169	CMR900	28989	12/2013	Eidolon helvum	Cameroon
KY770860	Jiyuan-84	29651	2012	Rhinolophus ferrumequinum	China
KU182965	JPDB144	30321	12/2012	Myotis daubentonii	China
KU182964	JTMC15	28761	08/2013	Rhinolophus ferrumequinum	China
KX574227	PREDICT/PDF-2180	29642	02/20/2013	Pipistrellus cf. hesperidus; specimen voucher: OTBA03-20130220	Uganda
NC_034440*	PREDICT/PDF-2180	29642	02/20/2013	Pipistrellus cf. hesperidus; specimen voucher: OTBA03-20130220	Uganda
LC469308	Vs-CoV-1	29930	-N/A-	Vespertilio sinensis	Japan
GU190215	BtCoV/BM48-31/BGR/2008	29276	2008	Rhinolophus blasii	Bulgaria
NC_014470*	BtCoV/BM48-31/BGR/2008	29276	2008	Rhinolophus blasii	Bulgaria
